# Role of climate change in economic uncertainty of Pakistan: New approach with qualitative comparative analysis

**DOI:** 10.1016/j.heliyon.2024.e40889

**Published:** 2024-12-05

**Authors:** Usama Usman, Xueyan Yang, Muhammad Ismail Nasir

**Affiliations:** a School of Public Policy and Administration, Xi'an Jiaotong University, Xi'an, Shaanxi, PR China; b Foundation University, Islamabad, Pakistan

**Keywords:** Climate change, Economic abnormality, Qualitative comparative analysis

## Abstract

Globally, the effects of climate change are becoming more pronounced. Simultaneously, concerns associated with climate change effects have garnered widespread attention. The motive of this study is to know about the prominent antecedents of climate abnormalities in Pakistan, which may lead to economic abnormality and instability. The core objectives of this research are to: identify the abrupt changes in the climate of Pakistan, know about the level of disruption towards economic conditions due to climate change, detect the aggregate consequences of climate change on the economy of Pakistan, and finally take steps to hedge the abnormalities resulting from the abnormal climate changes. The accomplices employed qualitative methods to gather information. Fuzzy set Qualitative comparative analysis (fsQCA) and semi-organized talks to thoroughly separate each plaintiff's extensive knowledge and opinions. For this study, a sample of 30 experienced economic analysts and climate change experts from Pakistan were chosen. The study's findings verified that several important antecedents, including the nature of climate change abnormalities, are identified in the research. This study explored methods to address these abnormalities and was conducted with professional guidance to meet sustainable development goals related to climate change. Scholars and experts are advised to adopt more systematic approaches to mitigate the risks due to the complex and variable combination situations that led to the climate change risks in Pakistan and the surrounding area.

## 1 Introduction

Climate change shifts the environmental pattern due to natural phenomena or human intervention. Changes in temperature, precipitation, pressure, and humidity may indicate climate change and global warming [1,2]. It is critical to comprehend the impact of climate on economic value to develop effective mitigation and adaptation policies [3]. It is however difficult to quantify the costs of climate change to the economy. The recent studies are focused on the statistical approaches using historical data as compared to the past studies that focused on process models to estimate the financial losses brought on by climate change [4,5]. The effect of weather variations cannot estimate the impact of climate change, its repercussions and adaptation are different from short-term and long-term changes. Climate change has welfare consequences in terms of weather realization and production choices [6]. Climate change's net economic price includes mutual adjustment and equilibrium costs. Because of the essentially inferior outcomes under the altered environment and because agents are first unprepared for or even aware of the altered climate, climate change lowers welfare. The remainder of the well-being loss, including the cost of adaption following adjustment to the new environment, is referred to as the balance or equilibrium cost of climate change [7].

Climate change is a global issue and has become a critical cause of concern for nations [8]. Due to the growing urgency of combating climate change, many advanced countries are also grappling with environmental deterioration, excessive utilization of energy, and greenhouse gas emissions [9]. South Asian countries especially developing countries like Pakistan are more vulnerable to the changes in climate and the awareness related to adaption and mitigation is very low [10]. Developing countries like Pakistan are more prone to the problems caused by climate change. The poverty and resource paucity of developing countries put them at a greater risk of victimization as compared to the advanced countries that have a stronger capacity to adapt [11]. Pakistan's increased levels of poverty and lack of food security are due to climate change and impact the largest economic sector of Pakistan [12]. Although the country is not largely responsible for greenhouse gas emissions, it experiences significant effects of change in the climate. It seriously jeopardizes every aspect of environmental sustainability.

Global warming and climate change also impact several socioeconomic factors [13]. Natural calamities and soaring temperatures cause health problems among individuals, may act as an obstacle to education, and cause unemployment due to migration. Moreover, it adversely affects the agriculture sector crucial to the economy [14]. Adverse effects of climate change include raised temperature, changing rainfall patterns, droughts, and water reserve shrinkage. Moreover, it increases water scarcity, food insecurity, and health issues in individuals. Climate hazards like floods and droughts may cause people to be displaced. People may lose their jobs while moving from one place to another. Food prices may rise due to scarcity of food, leaving many people vulnerable to food crisis and hunger [15]. These may result in issues like unemployment, inflation, and food poverty and eventually cause a decline in economic development [16].

The adverse effects of climate change including food poverty, water shortage, energy crisis, raised temperature, and related issues directly impact the nation's expansion of its economy [17]. The negative outcome of climate change also encompasses forestry, animals, aquaculture, and farming which are pivotal pillars of the Pakistani economy. Climate change will have a significant negative impact on Pakistan due to its reliance on agriculture [11]. Any change in temperature and rainfall patterns impinge upon the food security of the population. Being an agro-based country, low agricultural productivity translates into lesser economic output for the country. Moreover, our industries depend on the agriculture sector for raw materials, so lesser agricultural production may disrupt the supply chain [18]. Human progress, particularly economic activity, well-being, and access to energy, is impacted by the substantial effect that humans have on the natural environment. Therefore, to address more and more pressing climatic and environmental concerns, both industrialized and less developed nations globally have implemented a variety of policies, including reducing greenhouse gas emissions, preserving the environment, and speeding up transitions to cleaner energy [19]. The cost of these issues further stresses the country's economic position. Furthermore, the costs incurred on coping strategies and adaption measures are to be considered as well for effective development plans.

The paper adds in different ways to a deeper analysis of the issues raised by climate change. The study's primary focus is on professional, in-depth analysis of experts' interviews, as opposed to observational and conceptually inductive reasoning, which produces more objective and significant conclusions. Additionally, the QCA methodology was used to evaluate the opinions of experts on different issues. This method integrated the examination of numerical data with a qualitative inquiry, making it more complex and believable than previous studies on the same subject. Extensive interviews were conducted to gather the essential data for the qualitative evaluation to better understand the interplay of factors and causes of climate change. Since this is a complex subject with several facets, this article focuses on how, among other environmental causes, climate change leads to abnormalities in various economic variables as a result of the country's falling economy. It begins with an explanation of the variables causing climate change. These components of climate change that affect the economy directly or indirectly are the main focus of this study.

The study is organized in such a way. Section 2 discusses the literature review, Section 3 explains the data and methodology, section 4 is the results and discussions and the final section is the conclusion.

## 2 Literature review and theoretical backgrounds

Climate change has a momentous impact on populations, communities, and the performance of financial prudence. Recently, structural changes have drawn interest from several nations as a crucial means of achieving greenhouse gas reduction objectives. Noteworthy structural changes in energy, commerce, and societal life have resulted from the emergence of sustainable energy, which mostly consists of energy from renewable sources, the ongoing promotion of globalization to trade openness, the flourishing of the service and manufacturing sectors, and the growth of urbanization [20]. Climate factors like temperature have a huge impact on societies, heat induces mortality, and provokes aggression while reducing productivity. In addition to harming crops and raising electricity demand, high temperatures can lead to population shifts both inside and across international borders [8,21]. Tropical cyclones diminish economic output for extended periods, cause property damage, and cause fatalities. The trading patterns may also be influenced by the climate. In this regard, typhoon hits reduce government revenue, frequently decrease imports, and extreme heat, which lead to inefficiency, and reduce the number of products produced by a region, both in the agricultural and industrial sectors [6].

The frequency of both natural and environmental catastrophes can vary greatly from season to season; some years may have very few fatalities until a major catastrophic event that takes multiple lives occurs [6]. During the previous ten years, catastrophic weather events have claimed the lives of almost sixty thousand people annually on average throughout the world [22]. Many plants and other species are projected to perish as a result of climate change brought on by a lack of natural assets, a rise in the melting of glaciers, and increasing water levels [18]. There is a good chance that the current trends of rising temperatures, breakouts of bug diseases, health issues, and a shift in seasons and routine behaviors will continue in the years to come [12]. Around the world, both human and natural environmental disasters have resulted in enormous losses, including reduced agricultural yields, system restoration, and the reconstruction of essential technology [23].

Climate change has a substantial influence on macro-economic variables too [24]. Financial indicators and macroeconomic variables have a critical part in a country's economic volatility [25,26].talked about the financial and macroeconomic factors and their effects on the stock exchange. The fluctuation in the stock prices can be the result of the disparities in the macroeconomic factors that might affect not only the stock market but the dividends and so many other factors. The skilled and rich financial markets can likewise be highly beneficial to the country's economic performance management. These fluctuations can have a consequence on cash movements, which constitute the foundation of almost any financial market [27,28]. analyzed the variations among the capital flows and the exchange rates. In established markets, where factors do not fluctuate significantly, it would be easy to manage the capital flows in that market. In some kinds of marketplaces with large volatility of factors, there will be price/return confusion, and consumers will be hesitant to spend their funds in the market.

These variations can also be the cause of inflation in the country [29,30].talks about inflation persistence and exchange rate regimes. The variation of macroeconomic indicators can also have a significant influence on overall stock exchange market performance, and investment returns influence the 100 indexes [31,32].described the influence of macroeconomic factors on aggregate returns of the stock exchange marketplace. There exists a significant connection between macroeconomic variables and the stock exchange marketplace. The impact can be positive as well as negative [33,34].will look into the relationship between macroeconomic indicators and stock values. The prices of the stocks would vary automatically on the market as the macroeconomic variables fluctuated, such as changes in oil prices and currency rates, which typically have an impact on any economy, particularly those in developing nations [35].

According to the new research [36,37], climate change affects the economy on a macroeconomic level. Tropical cyclones are said to have a linear relation with economic growth and they may result in slowing down of GDP growth depending upon the intensity of the storm. Moreover, the temperature has a non-linear influence on productive capacity is so significant that output is greatest at about 13 °C. Heavy precipitation hurts businesses and communities, this is more evident in the agriculture-based surroundings. These impacts are frequently quantifiably substantial. It is predicted that upcoming heating can sluggish the development by 0.28 %. Climate change may also result in demographic distortions [36]. Development in the economy and productivity in general are strongly influenced by the climate. As a result of its impact on economic development and growing worldwide presence, climate change has emerged as a top priority for national and international ecological authorities [38]. Thus, it is important to comprehend how climate change affects the agriculture sector's total production factor when developing regional adaptation plans and structuring effective climate policy agreements. The effects of global climate change on the agriculture industry were previously predicted by earlier research. According to research, different parts of the world will be affected by global climate change in the agriculture industry. Scientists' main focus now is on analyzing how climate change affects different agricultural activities in different geographic areas and developing appropriate responses to its consequences [39].

According to recent empirical data [40,41], modern people are already under tremendous economic and social pressure as a result of the existing environment, and future climate change will only make these costs rise much more [40]. Theoretically, losses in the now and the future may be prevented if communities were to properly adapt to climate change. When these calamities occur, populations may adopt measures or make investments that will lessen their impact. To decrease the impacts of global warming and climate change, the Ministry of Climate Change of Pakistan has taken several actions [42]. The main causes of heightened climate change consequences, however, are an absence of consciousness and understanding about operative actions, weaknesses in organizational capabilities, a lack of resources and their inefficient use, and poor economic conditions. Pakistan is making efforts to reduce carbon emissions and improve the environment quality through obtaining cash through the Asian Development Bank's (ADB) global climate finance. Also, the Green Pakistan Program is being conducted throughout Pakistan.

The two most vulnerable industries are those related to water and agriculture. Rainwater harvesting, stormwater management, and groundwater recharge are the three technologies for the water industry. The agriculture industry's preferred technologies include effective irrigation systems (both drip and sprinkle), crops that can withstand drought, climate estimations and forecasts, as well as the presence of an early-warning system [43]. To tackle the ramifications of the changing climate, Pakistan needs to focus on drafting national development policies and plans that are effective in dealing with issues related to the economy and society along with the issues in the priority sectors. Both the Kyoto Protocol and the United Nations Framework Convention on Climate Change include provisions for policy frameworks that should be used to drive the Climate Change Action Plan [44].

This research is different from other previous studies’ literature as it uses qualitative interview surveys method and Qualitative Comparative Analysis to assess the economic abnormalities and their drivers because of climate change. This study adds a new theoretical foundation of Qualitative Comparative Analysis with Qualitative Analysis to analyze the drivers and impacts of climate change which previous studies have not used.

## 3 Data and methodology

### 3.1 Data overview

For this study, the climate change experts and economic analysts from Pakistan are the population. Semi-structured discussions go with abstract, all-out material. Besides, for this study, thirty interviews were systematized (Table 1) – and the majority of candidates provided thorough and clear responses by the study's restrictions. From February 2023 to April 2023, there were thirty distinct days during which the interviews were done. Thirty comprehensive semi-structured interviews were performed overall, with inhabitants of Islamabad city participating in fifty percent of them and non-residents of Islamabad city participating in the remaining fifty percent. In all, there were 30 % women respondents and 70 % men respondents. Surveys with stakeholders, such as government workers, officials, members of the media, and staff members of non-governmental organizations (NGOs), were also undertaken.

**Table 1:** Interview respondents.

Job role	Pakistan
Category	
Economic Analysts	16
Climate Change Expert	14
Total Participants	30

Table 1's job descriptions demonstrate that all of the candidates have a broad understanding of climate change and economic irregularities.

### 3.2 Sampling technique

Qualitative or mixed study design frequently depends on participants who can communicate and reflect well enough to offer detailed accounts of what they've experienced. Interviews with uninterested participants that yield vague replies are not good for analysis. Smaller sample numbers and realistic observation and interviews are preferred by qualitative approaches. Survey interview research design has been employed by us for both the data collection and analysis. we have employed a purposive sampling method, because of the respondents' specific characteristics which are significant in a group, financial resources, time limits, travel expenses, and other logistical issues related to in-person interviews. A purposive sample consists of persons who happen to be the most relevant, approachable, and perhaps able to give the scholar the details they require. We have conducted the survey interviews with economic and climate change experts in Pakistan using purposive sampling [45].

The primary interview questions were derived from the body of work already published on economic irregularities and climate change. Table 2 includes the interview questions. The interviews are currently underway, and they cover a wide range of topics, including climate change and macroeconomic fluctuations, climate change and consumer responses, climate change and economic abnormalities, and controlling perspectives toward climate change abnormalities. The interviews then delved deeply into the specific topics covered in the literature. The one-on-one conversations often lasted between 50 and 60 min and even up to 75 min for each person.

**Table 2:** Interview details.

**Interview protocol**
The interview processes
Inform the interviewer(s) and applicant(s)
Plan your research strategy.
Plan the study's grit, taking the goalposts into account.
In opposition to potential research worries, moral subjects, and reaching a consensus
Prepare for the interview or focus group in advance.

### 3.3 Research themes and specific questions

#### 3.3.1 Climate change and individual responses

1. To what extent, is an individual's thinking about (harsh weather conditions, greenhouse gasses, industrialization, energy consumption) towards climate change?
2. How to control the antecedents of abnormal climate changes?

#### 3.3.2 Climate change and macro-economic fluctuations and economic abnormalities

1. What are the main types of climates that may present in Pakistan, and what are their effects on the macro-economy (poverty, fatalities, financials, businesses, unemployment)?
2. May you please share the different effects of climate change on the economic variables (transportation system, infrastructure, migration, crops, food insecurity, clean drinking water, decoupling)?

#### 3.3.3 Controlling perspective for climate abnormalities

1. What to do towards hedging the abnormal conditions of the climate?
2. What role do you play in these sorts of conditions?
3. What are the effects of your part in definite situations?

#### 3.3.4 About experts

1. What kind of information is vital for you in the direction of analysis/advice at a time of uncertainty concerning the economy?
2. What do you see as the key controls to do hedging with cognizance?

### 3.4 Variables description

Because of the multiple risk variables and circumstances in the climate change auditing report, this study used the common auditing qualitative description to ensure the logic and correctness of the QCA outcomes. Later, seven classifications and types of those discovery hazards were established by the Pakistan auditing common qualitative statements, relevant rules, and financial audit. Even more significant, every categorization, to certain degrees, describes the precise appearance and issue features. The comprehensive assessment, induction, and categorization that make up these seven classes are appropriate as prerequisites for QCA analysis, therefore all seven of them would be chosen as requirements for climate change reporting hazards via auditing shown in Table 3. The results of the QCA investigation would determine the percentage of illegal spending because it offers a collection of instruments for analyzing the required and sufficient circumstances, demonstrating outcomes, and connecting parallels and discrepancies between different combinations of situations and scenarios. The threat associated with climate change increases with increasing values of indicators and decreases in spending efficiency.

**Table 3:** Variables description.

Determines	Name	Contractions
Events	Infringement of policy implementation regarding climate change	Pi
	Infringement of funds and resources	Fr
	Infringement of town planning	Top
	Infringement of laws for the preservation of historic sites and sustainability	Hs
	Infringement of infrastructure in Riverland	RL
	Infringement of regulation of deforestation	Df
	Infringement of rules for quality control	Qc
Outcome	Percentage of illicit spending	Is

### 3.5 Methodology

A mixed research method strategy was employed since a single quantitative or qualitative approach was insufficient to comprehend and describe the study's concerns. The qualitative comparative analysis has been used for empirical analysis and the thematic analysis approach has been used for qualitative analysis. In this sense, a quantitative research approach was initially used to analyze the factors that drove. Then, via the use of the interview method, the associations between the variables in question were further investigated. The versatility of thematic analysis makes it appropriate for analyzing a broad variety of data sources. For example, data from "standard" in-person data-gathering techniques like interviewing and conducting focus groups may be investigated using thematic analysis. This research project therefore exemplifies a descriptive mixed-technique approach.

The application of qualitative comparative analysis (QCA) in management research demonstrates that despite the intricate nature of the field of management issues, there are seldom explored growth avenues that may be revealed through study. As a result, QCA can enhance knowledge about increasing management issues while maintaining their comprehensive character [46]. Considering the aforementioned reasons, this study used the QCA approach, which could bring together the benefits of qualitative and quantitative analysis, to comprehend the influencing variables and creation mechanisms of climate change threats through expert opinions. Even though this study incorporates the opinions of various experts, the scenario's study is unable to achieve the standards of a big sample, making it difficult to obtain trustworthy outcomes using statistical tools. QCA is excellent at limited sample assessment within 10 and 40 cases for an in-depth comprehension of a real event with an integration of quantitative statistical analysis and qualitative analysis [47].

The purpose of the QCA technique is to determine the connections between the contingent configuration and its outcome using case comparison, determining which contingent configuration will result in the anticipated result and which contingent configuration might result in if not present while taking the interrelationships of influencing factors into consideration. QCA techniques are potential instruments for bridging the divide between variable- and case-oriented investigation [48]. There are three primary QCA analysis techniques fuzzy set QCA, crispy set QCA, and multivalued QCA [49]. Of these, fuzzy set QCA is the most well-known and has been applied in several studies to date. We will use the fuzzy set QCA technique in the first analysis of this study.

The second analysis of this study relied on a subjective method (qualitative methodology) to thoroughly elicit the candidates' thoughts, as also used by Ref. [50]. Deep discussions (interviews) were performed to gather primary data for the thematic analysis to gain a greater understanding of how climate change and economic factors interact. Additionally, the qualitative thematic analysis was supplemented by the investigator's perspectives as well as information from relevant online platforms and firsthand witnesses. It is undeniable that neither some information nor an estimate on potential responses were previously given to the candidates. In this study, multiple interview data [51,52] was utilized to examine the relationship between climate change and economic factors. Numerous examples make it possible to classify configurations and fundamental overtones using a practical inspection of the topics and signals.

The key debate points centered on the study's primary research questions: herding bias and irregularities in the economic factors. This study was able to identify several open crow's nests, allowing a proportional downfall of maneuvers to the application of analysis (based on a high degree of awareness) immediately before dealing with the issue in which stockholders invested due to herding bias and a lack of market expertise.

## 4 Estimation results

To create intricate databases utilizing rational and comprehensive methods, QCA bases itself on the Boolean algorithm, which permits the simplest formulae and whose set of conditions and results have values that are either 0 or 1 having variable segments. The infringement of policy implementation regarding climate change is 76 percent and has a value of 1 on the other hand no infringement of policy implementation is 24 percent and has a value of 0 in Table 4. Similarly, this table shows the occurrence and non-occurrence of all the variables in percentages and with the values of 1 and 0.

**Table 4:** QCA variables and their segments.

Variables	Determines	Portions	Value
**pi**	Occurred	76 %	1
	Not Occurred	24 %	0
**fr**	Occurred	56 %	1
	Not Occurred	44 %	0
**top**	Occurred	83 %	1
	Not Occurred	17 %	0
**hs**	Occurred	70 %	1
	Not Occurred	30 %	0
**RL**	Occurred	90 %	1
	Not Occurred	10 %	0
**df**	Occurred	73 %	1
	Not Occurred	27 %	0
**qc**	Occurred	53 %	1
	Not Occurred	47 %	0
**is**	Occurred	53 %	1
	Not Occurred	47 %	0

### 4.1 Results and discussion of fsQCA

The fsQCA 3.0 software was used to conduct the analysis. The circumstances and results were calibrated in the first stage. When using fsQCA, calibration is required. Calibrating requires the definition of three observation points: 0.05 for complete non-membership to the set, 0.5 for the point of greatest uncertainty, and 0.95 for complete membership to the set. It is necessary to calibrate the system before building the truth table, which will yield distributions of possible outcomes for each possible set of conditions. FsQCA allows researchers to find several paths to a solution.

The fsQCA's intermediate solution is displayed in Table 5. The approach logically reduces the configurations using the Quine-McCluskey algorithm. The membership in each configuration affects how much the configurations' means for the result are weighted. The mean, weighted by the highest value of the other configurations, is tested, and this value is published against it. The n consistency of each configuration (inclusion in not − y, or 1 − y) is compared to the y consistency of each configuration (inclusion in y). Results that are not significant (at the 0.1 threshold) are excluded. This approach necessitates deducing the predicted contributions of each causal set to the outcome.

**Table 5:** fsQCA Results.

Paths	QC	DF	RI	HS	PI	FR	TP	Raw Coverage	Consistency
Qc∗Df∗Rl∗HS∗Pi	●	●	●	●	●			0.564329	0.842105
Df∗Rl∗HS∗Tp∗Pi		●	●	●	●	●		0.276515	0.863636
Qc∗Df∗HS∗Fr∗Pi	●	●		●	●	●		0.261021	0.833331
∼Qc∗Df∗Rl∗HS∗Tp∗∼Fr	○	●	●	●		○	●	0.481402	0.772942
Qc∗Df∗Rl∗Tp∗∼Fr∗Pi	●	●	●		●	○	●	0.521455	0.983644

Note: ● represents the presence of a condition and ○ the absence of a condition.

There are three options: both presence and absence. (Ragin, 2006) proposes a bottom bound of 0.80 for a high score in the result. As a result, we eliminated any solution with a consistency of 0.80 or below. Assumptions made for the parsimonious solution may not be valid. Thus, we computed the intermediate solution. Counterfactuals are used in intermediate solutions to reduce the complexity without relying on erroneous assumptions. This process necessitates considering each causal set's predicted contributions to the result. There are three options: both presence and absence. The truth table's solution term (Table 5) illustrates the connection between several sets of criteria and the result. The combination of criteria suggests a favorable association between climate change and the economic condition of Pakistan. The results show that all the variables are good and consistent because variable consistency is above 0.74. Whereas, raw coverage should be 0.25 to 0.65. Raw coverage results also lie in the standard range. So, we say that all the variables have good raw coverage. In necessary conditions Table 6, all the variables are consistent and covered. This approach is an intriguing exception to the general tendency, covering 3.8 % of the sampled firms. This route may be related to economic growth and includes government and policymakers to improve the climate condition of the country.

**Table 6:** Analysis of necessary conditions.

Variables	Consistency	Coverage
PI	0.860320	0.327586
FR	0.769393	0.479166
TP	0.880466	0.388024
HS	0.947248	0.285185
RI	0.960300	0.392856
DF	0.921034	0.289285
QC	0.760002	0.582608

The study found that climate change hazards in Pakistan were not triggered by one specific factor, but rather by a complex combination of factors (Pi, Fr, Top, Hs, RL, Df, Qc). Configuration assessment is a novel kind of research tool that examines the internal workings of climate change hazards and comprehends their microoperation method; as a result, this study will examine the risks associated with megaprojects by evaluating and classifying eight criteria in conjunction with the pertinent specifications. Seven configurations were then compiled using QCA. This statistical approach can help the industry strengthen its risk-control measures.

### 4.2 Results and discussion interview-based analysis

Thematic analysis was conducted on transcriptions of the interviews following the themes identified using qualitative and quantitative data analysis. The same were identified through simple observations manually from transcriptions of the interviews. Numerous items might be referred to as "thematic analysis," including yet but not restricted to social sciences data assessment methods. Thematic analysis is a technique for discovering patterns in qualitative research that is often used today. It has also been asserted that thematic analysis evolved from the study of content analysis, and the terms "thematic analysis" and "content analysis" are frequently used consistently to describe both qualitative as well as quantitative analysis [53].

We have used five phases of thematic analysis in our interview analysis. Familiarizing oneself with the information is the initial step in the thematic analysis procedure, which we had started at the time of gathering the information. To get more involved the researcher in the information and provide the foundation for analysis, the next step is creating codes. As the coding process went on, we began to identify commonalities and trends within the information being analyzed. Before transitioning from coding to thematic construction in the third stage, it is critical to maintain emphasis on processing the complete data information. In the fourth stage, the ideas we generated were fluid and subject to change, much like the initial version of an original piece of work. The fourth step was to evaluate prospective themes. Afterward, in the fifth and last stage, creating the report after generating the full analysis. The interviews drafting and the thematic assessment of the information is written below.

“I have never seen climatic carnage on the scale of the floods here in Pakistan”, remarked a climate change expert during the interview. All nations will experience losses and damage from the climate as our world continues to warm beyond their capacity to adapt. This is an international crisis. It needs an international response. Floods in 2022 and an exceptional stretch of torrential rains in recent months, as well as the loss of some species and the extraordinary melting of glaciers, are all warning signals of what is to come. (Interview)

The concentration of greenhouse gases (GHGs) is constantly exceeding new thresholds, according to climate change advocates. The primary factor causing the global climate change is GHGs. Devastating natural tragedies are one way that this change manifests. A subsequent climate change excerpt who is working on GHGs in Pakistan makes the idea clear: “Pakistan is responsible for the majority of the repercussions while producing only point three percent of the world's GHGs by volume”. A large portion of GHG emissions is produced by the US, China, and India. (Interview)

The quote that follows is particularly revealing with facts given by an expert. The conflict between financial expansion and carbon dioxide gas releases is another one of the century's biggest problems. The emergence of the industrial revolution from the nineteenth to twenty-first century has resulted in increased GHG emissions including atmospheric CO2 by almost thirty-five percent. “If the GHG emissions are not controlled, a rise of about 1.4–5.8 °C is expected in the global warming”. (Interview)

Pakistan is among the region's most negatively impacted by global warming, and climatic changes and is ranked 16th in the vulnerability index. As a result, the country faces a lack of clean drinking water & food insecurity. An expert explained that: “The significant increase in the GHG emissions results in Climatic changes which carriage a great danger to Asian countries like Pakistan, India, and China”. The population and energy intensity are the main factors influencing ecological excellence in Pacific Island Countries (PIC), according to the beliefs and knowledge of the experts. The respondents also show that in PIC countries, population growth and prosperity both worsen environmental quality by raising CO2 emissions. However, relative to Pakistan, India, and China, China is more negatively impacted by affluence. Energy structure and carbon intensity had a conflicting impact in PIC countries; that is, in some years. They increase ecological excellence while in the remaining study years, they degrade it. However, the most important element that has a direct impact on how much CO2 is emitted in PIC countries is energy intensity. (Interview)

China is the biggest emitter of CO2 and the biggest energy user on the planet. The emissions have grown at an exponential rate. A viable choice to reduce CO2 emissions is to limit energy consumption. However, China uses over sixty-nine percent of the total of the world's energy, so any efforts to cut back there would have a similar effect on the country's economic growth and global trade. India is industrializing at a fast pace but not without a cost. The country faces many environmental issues including degradation of air quality, disruption of coastal ecosystem, natural disasters like floods, and unexpected weather events. Malnutrition, disease exposure, lost revenue, and destroyed livelihoods are a few ways that such calamities hurt the region's economy. (Interview)

Moreover, the expert's analysis revealed that Pakistan underwent expensive coupling, weak economic decoupling, and strong decoupling throughout the investigation. In General, Pakistan witnessed exclusive negative decoupling, meaning that increases in CO2 emissions reflect the country's economic growth. “In addition, significant decoupling also took place during 1991, 1995, and 2009. India went through tetrad decoupling phases: exclusive coupling, expensive decoupling, expensive negative decoupling, and expensive robust decoupling. Generally, India shows feeble decoupling, which means that its rate of financial performance is more advanced than its rate of increase in CO2 releases. Additionally, India also showed exclusive coupling, which indicates that these years did not see any decoupling, whereas robust decoupling solitary happened from 2010 to 2020”. The results of the expert's analysis show that China exhibits feeble decoupling over the majority of the research period, as well as exclusive coupling and exclusive negative decoupling. The level of energy increases the decoupling progress in PIC nations, according to the expert's analysis. Although the energy structure and CO2 emissions have conflicting effects on the evolution of PIC's decoupling, they occasionally promote decoupling while occasionally impeding it. Similar to this, population growth and wealth both contribute to the slowing of the decoupling process in PIC nations. (Interview)

**Fig. 1:**
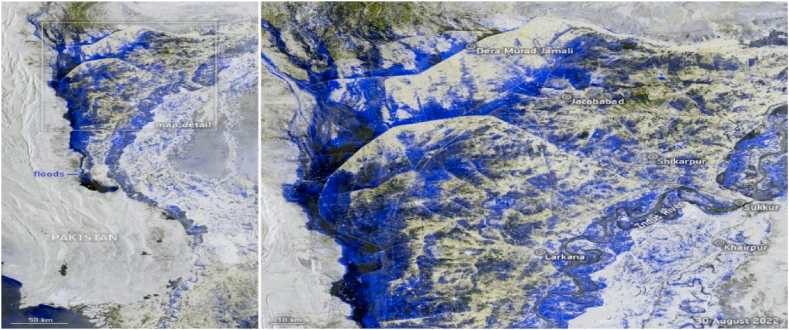
A photograph of Pakistan taken from space on September 30, 2022, shows the country submerged in floodwaters. By Sentinel-1, a European Earth observation satellite.

Despite its small contribution to global carbon emissions, Pakistan is experiencing the effects of climate change. Due to its geographical location and diverse tropical continental climate, the nation experiences severe climate-related natural hazards. Residents of the impacted areas have been devastated by the recent floods. Take note of the information an expert member stated: “By September 19, the floods had impacted about 2 million homes”. (Interview). Fig. 1 shows the massive scale of floods in the country recently has flooded 25 % area of the country.

According to the writers' research, expert assessments, and investigations, environmental occurrences that contribute to macro-level economic losses are projected by quantified numbers. Interviewees explained macro-economic losses by sharing some facts. Climate change has contributed to an increase in poverty and unemployment. The economic experts projected Pakistan's poverty level to be 39.3 % by using $3.2 per day lower middle-income poverty criteria. For the fiscal years 2020–22, the rate for the upper middle class is set at $5.5 per day. This demonstrates how severe the economic crisis is in Pakistan. Pakistan is ranked sixth globally in terms of the Global Climate Risk Index. “Between 1999 and 2019, Pakistan had 91,089 fatalities, $81 billion in economic losses, and 152 instances of extreme weather”. (Interview).

The main area of climate change that affects Pakistan is its water cycle according to interviewees. One of the key industries most likely to suffer from climate change is agriculture. Quality of food, accessibility, and supplies are all impacted by climate change. The performance of agriculture may be impacted by projected rises in temperature, modifications to rainfall patterns, modifications to severe weather events, and reductions in water supply. “Climate change and pollution are also the causes of seasonal smog”. (Interview)

Several respondents stated people currently must deal with new problems such as food insecurity and clean drinking water on top of the two difficulties of unemployment and poverty brought on by climate change. Additionally, certain respondents mentioned that despite that they had already come into contact with food and drinking water, they had noticed a huge rise in both of these problems in recent years to the point that they are now among the top concerns for the public and government. (Interview). Numerous areas have lost significant crops and sources of income. The nation's food security is now seriously threatened as a result of this. Pakistan needs to import food even though it is an agricultural nation. A number of the poll participants made the observation that increased rain and flooding can disrupt the systems for distributing and transmitting electricity. This frequently results in a daytime interruption in electricity, which negatively affects daily living, and businesses and eventually raises general disappointment. Foreign exchange reserves and businesses decrease as a result. (Interview)

**Fig. 2:**
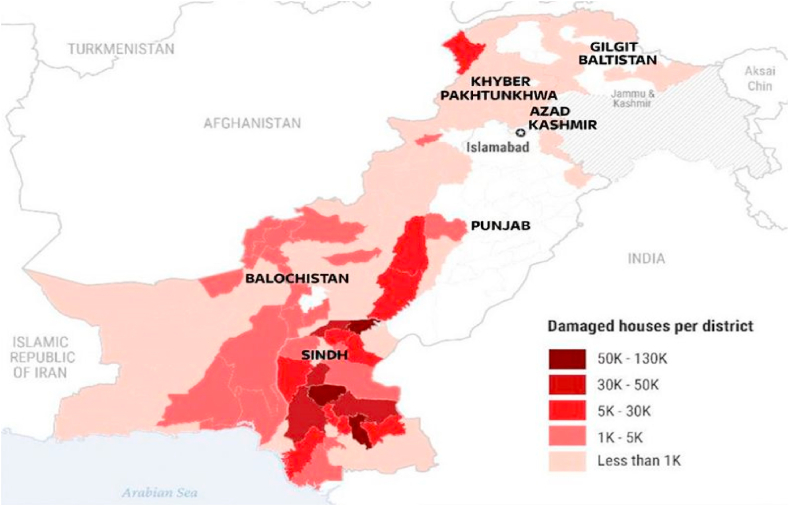
A United Nations (UN) visual shows the magnitude of the destruction of properties throughout Pakistan.

Participants in the interview discussed how the environmental catastrophe brought on by climate change has affected towns as well as rural infrastructures, notably damages to roads and schools. “Roads totaling 12,700 km were damaged, and 7.6 million people were directly impacted. Around $30 billion has been calculated as the total loss. In Baluchistan, Sindh, and the Punjab, more than 80 districts were submerged. The school system has been badly impacted, along with other industries. The exceptional rains damaged or destroyed 17,566 schools, including 1584 in Baluchistan, 1180 in the Punjab, and 15,842 in Sindh”. (Interview). According to some respondents, environmental issues including infrastructure deterioration and climate change-related factors like rising temperatures have put the homes of the people in danger, which has forced them to move. As a result, a sizable portion of the populace has crossed the danger boundary and has reacted to the shift by migrating. (Interview). The extent of the devastation to dwellings around Pakistan is depicted in Fig. 2. We can see the infrastructure damage including houses all over the country particularly in the provinces of Sindh and Baluchistan.

Several respondents recommended that a bad economic catastrophe can be avoided by implementing a few wise and useful policies. To stabilize the economy, measures should first be implemented to boost exports from the nation. A specific focus should be given to political and economic issues including reducing obstacles to foreign direct investment. To improve domestic output and employment, special measures should be taken to win over foreign investors. It is important to start implementing specific plans as soon as feasible to capture renewable energy sources. “The use of solar and wind energy can help meet the world's energy needs”. (Interview)

The participants emphasized that Infrastructure that is climate resilient needs to be prioritized. The nation urgently needs to invest in its human resources. “One of the youngest populations in the world is found in Pakistan”. It is past time to involve Pakistan's youth in the construction of infrastructure that is climatically resilient. Controlling unauthorized building construction beside rivers and streams should be the responsibility of the competent authorities. It is important to educate the public on how to deal with natural disasters to reduce losses. Budgets should be set aside by the government to deal with unforeseen disasters to minimize the loss of life and property. (Interview)

Experts incorporated that as well, people's incomes, health, housing, infrastructure, and food security are all at stake in response to climate change. To lessen the impacts of climatic risks, the government should follow worldwide best practices. To lessen the effects of climate change, cooperation with international organizations should be improved and significant action should be taken. Fluctuations, earthquakes, and storms can all be lessened by taking precautions. “Long-term precautions against natural disasters include the construction of dams and water reservoirs”. The regional development strategy ought to include public input regularly. One of the senior interviewees expressed, that Pakistan should insist that the UN and its related organizations give the development of fundamental infrastructure significant consideration. The public should be made aware of the risks posed by climate change through awareness campaigns so that the average person may contribute to mitigation measures. (Interview)

Several economists stated Pakistan must increase foreign direct investment (FDI) to fund resilient and sustainable development initiatives and to boost the businesses in the country. To combat the poverty brought on by climate change, the government should take some unique measures to strengthen small farmers, women, and laborers. Loans and small- and medium-business initiatives may be among them. Delinking economic expansion and environmental output is of immense importance to achieve sustainable economic development. PIC countries use around twenty-nine percent of the total energy produced worldwide. “The fast-paced economic growth of China impacts its neighboring countries”. Pakistan should demand that China reduce its omissions and also pay the cost of economic damages in the region. (Interview)

Additionally, during the discussions, fresh factors for an environment that were rarely mentioned in earlier studies were revealed. Persons who are impoverished and disadvantaged are advocating for even more significant climate change action. Climate change is not only a disaster for the ecosystem; it is also a societal issue that compels us to tackle injustice on numerous other levels, such as that between men and women, decades, and rich and poor nations. “For more efficient development outcomes, the International Panel on Climate Change (IPCC) has underlined the requirement for reducing carbon emission that adheres to the principles of environmental justice (i.e., recognition, procedural, and distributive justice)”. (Interview)

Furthermore, a number of those interviewed held the belief that communities provide a variety of perspectives, and expertise, to the problem of boosting resistance and battling global warming. They must be viewed as partners in resistance development instead of recipients. Climate change is primarily caused by human activity. Climatic change is the primary cause. Burning of fossil fuels such as oil and coal has led to a rise in the quantity of carbon dioxide released into the atmosphere. There has been an increase in global heating as a consequence of the greenhouse effect's spread. “This phenomenon can be attributed to the fact that some chemicals in our atmosphere, including water vapors, carbon dioxide, methane, nitrous oxide, and chlorofluorocarbons, block heat from leaving the planet's surface, thinning the ozone layer and raising temperatures”. (Interview)

Another significant problem in Pakistan's industrialized eastern Punjab region is smog, which causes Lahore, the province capital, to become heavily polluted throughout the winter mentioned by interviewees. Authorities claimed that they are attempting to address the issue, which affects a large number of brick kilns. “Furthermore, heat stroke, starvation, the rise of vector-borne diseases like dengue virus, a rise in a load of water diseases, and other factors will affect people's capacity to work and make a living”. Moreover, multiple individuals referred to deforestation and a rise in the usage of pesticides in home and agricultural settings are two additional climate change-related factors. The second largest contributing factor to global warming. Deforestation is responsible for about twenty-four percentage points of all emissions of greenhouse gases. (Interview)

## 5 Conclusion

We have been the first to discover climate change's multiple drivers and various economic abnormalities associated with it by qualitative comparative analysis and the last ones to have such an opportunity to prevent it from happening, making it the most important issue of our time. In this study, we used qualitative analysis to identify several elements of climate change and their influence on various economic variables, and we used QCA analysis to analyze certain variations. Hazards associated with the causes and effects of climate change are rising along with its multifaceted nature. Authorities and a significant number of experts have realized that conventional investigation methodologies for identifying climate change risks have difficulty reflecting the scale of the issues, particularly routine evaluations of specific threats. Therefore, the related recommendations would have minimal impact on reducing the hazards associated with climate change. Accordingly, this study analyzes the hazards associated with climate change by evaluating and separating seven scenarios in conjunction with the pertinent expectations. QCA analysis provides an innovative form of study instrument that explores the inner nature of climate change difficulties and grasps their micro and macro action procedures. Seven variants were then compiled by QCA. The findings showed that complex and variable mixture conditions, rather than one specific factor, were to blame for Pakistan's climate change risks, which represented a significant advance in the field of quantitative and qualitative analysis as well as an organized strategy for the community to reduce climate change risk to a manageable level. Climate change's reverberating effect on various additional economic factors is more profound and multifaceted than its obvious effect. Furthermore, as the qualitative research shows, economic forces have a significant causal influence on other elements, particularly structural infrastructures.

### 5.1 Policy recommendations and way forward

The changing climate has put many countries in danger, and rising economies are particularly vulnerable. Living in a bubble of ignorance won't get us very far because the world is witnessing a melting glacier problem, rising floods, animal extinctions, extreme weather events, and much more. It is imperative to spread knowledge of climate change in every manner possible, even though seemingly worthless tasks like completing school assignments. This crucial issue, which is exerting a severe impact on the region, has made South Asia more susceptible to calamities. Pakistan is generally experiencing severe effects from climate change and global warming. The changing climate puts Pakistan's economy, real estate market, food production, and stability in danger. Given the stark realities, the Pakistani government should move swiftly to fight the harmful consequences of climate change. There is little doubt that the officials are paying attention to this matter since they view it as sensitive and significant. As the climate changes, millions of poor people will face serious issues like extreme weather, health effects, risks to heritage and culture, financial stability, transportation, water management, and social welfare.

As a result, productivity in the agricultural, manufacturing, and service sectors all exhibit negative and substantial relationships with temperature. If climate change is not handled, it will severely hinder economic progress. To deal with the influence of climatic changes on many sectors, adaptation and mitigation strategies are required at the micro level. Climate change, however, is a global problem. Pakistan, in comparison to affluent nations, contributes very little to GHG emissions, making it very difficult for Pakistan to mitigate climate change. Alternative energy sources are more effective and help solve the global warming issue. Power generation from sunlight, winds, tides, and biofuels is more environmentally friendly and sustainable. If we generate electricity using other energy sources, the effects are minimal. Nuclear power produces a small amount of greenhouse gas emissions; increasing its inclusion in the energy mix might aid in reducing global climate change.

Emerging economies must receive financial support from richer countries to switch to low-carbon development pathways and support them to become ready for the consequences of climate change in order so that there can be a sustainable global climate change accord. The main source of global warming is the energy required to operate, heat, and cool our homes, enterprises, and factories. Energy-efficient solutions are an immediate necessity. Essentially, we need to implement a double strategic plan: firstly, we should cut emissions and stabilize the levels of greenhouse gases in our atmosphere; second, we should adopt climate-friendly habits and uphold the principles of sustainable economic development.

### 5.2 Limitations

A new type of analysis tool called qualitative comparative analysis explores the internal workings of climate change risks and grasp their economic impacts. For this reason, this research analyzed the risks associated with climate change by auditing and classifying seven conditions (variables) along with the pertinent specifications. More circumstances (variables) for examination may be included in subsequent studies. The findings suggested that complex and variable combination conditions rather than a single factor contributed to Pakistan's climate change risks. Future studies can incorporate numerous identified factors into the study and analyze them. This would open up new avenues for assessing climate change risks using quantitative analysis methods and systematic thinking to help policymakers raise the risk-controlling threshold. We have collected information from 30 economic and climate change experts for survey interview analysis and QCA due to the limited availability of experts during our research. Future studies can use more experts' information for qualitative and QCA analysis to obtain more comprehensive results.

## CRediT authorship contribution statement

**Usama Usman:** Writing – review & editing, Writing – original draft. **Xueyan Yang:** Supervision. **Muhammad Ismail Nasir:** Data curation.

## Availability of data and materials

Data can be accessed with the permission of the author.

## Additional information (Ethical approval)

Foundation University Islamabad, Pakistan approved all experimental protocols (interviews surveys).

## Sources

Every source was included in the reference part and referenced inside the text.

## Funding

This paper has no funding.

## Declaration of competing interest

The authors declare that they have no known competing financial interests or personal relationships that could have appeared to influence the work reported in this paper.
